# The effects of beta2 adrenergic receptor gene polymorphism in lipid profiles

**DOI:** 10.1186/1476-511X-7-20

**Published:** 2008-05-21

**Authors:** Wei-Tsung Kao, Yung-Chieh Yen, For-Wey Lung

**Affiliations:** 1 Department of Medicine, Kaohsiung Armed Forces General Hospital, Kaohsiung, Taiwan; 2 Graduate School of Human Sexuality, Shu-Te University, Kaohsiung County, Taiwan; 3 Department of Psychiatry, E-Da Hospital, and College of Medicine, I-Shou University, Kaohsiung County, Taiwan; 4 Graduate Institute of Behavior Sciences, Kaohsiung Medical University, Kaohsiung, Taiwan; 5 Department of Psychiatry, National Defense Medical Center, Taipei, Taiwan; 6 Calo Psychiatric Center, Pingtung County, Taiwan

## Abstract

**Background:**

Explore the interaction between apolipoprotein E (Apo E), phospholipase A2 (PLA2) and β2 adrenergic receptor (β2-AR) gene polymorphisms and lipid profiles in an elderly Chinese population.

**Methods:**

Five hundred subjects aged 65 to 74 years were randomly selected from a community in southern Taiwan to assess the relationship between Apo E, PLA2 and β2-AR gene polymorphisms and lipid profiles. Two hundred sixty-seven participants agreed to have venous blood drawn for DNA studies.

**Results:**

Two statistically significant differences were noted in TC and LDL-C in the Gln27Glu of the β2-AR gene polymorphism (P = 0.007, P = 0.022). The low-income group had a higher HDL-C level (p = 0.076). The Gln27Glu polymorphism Glu/Glu or Gln/Glu subjects had lower TC levels compared to the Gln27Glu polymorphism Gln/Gln subjects (p = 0.092).     Lower TC levels (p = 0.082) and lower LDL-C levels (p = 0.045) in subjects with the Cys^19^Arg^16^Glu^27 ^haplotype. Lower TC levels (p = 0.06) were also noted in subjects with the Cys^19^Gly ^16^Glu^27 ^haplotype. On the other hand, higher VLDL-C levels (p = 0.185) and higher triglyceride (TG) levels (p = 0.190) were noted in subjects with the Cys^19^Gly ^16^Gln^27 ^haplotype. The ε2 allele combined with low income had a positive effect on HDL-C (p = 0.0011), after adding the income factor in this study.

**Conclusion:**

When the effects of Apo E and PLA2 on lipid profiles were included in this study, β2-AR gene polymorphisms reduced significant effect on lipid profiles. Similarly, low income increased effect on HDL-C. This study appeared that the results of gene-gene and gene-environment interaction, it should be considered in further studies for lipid profiles.

## Background

In a previous study, endogenous catecholamines were involved in the regulation of adipose tissue lipolysis, nonesterified fatty acid distribution, lipoprotein metabolism, obesity, glucose homeostasis, diabetes mellitus, blood pressure and metabolic syndrome [1]. Metabolic syndrome was defined as abdominal obesity, hyperglycemia, hypertension, hypertriglyceridemia and low high-density lipoprotein cholesterol (HDL-C). Adrenergic receptors (ARs) can regulate lipid mobilization, energy expenditure and glycogen breakdown through these endogenous catecholamines [1]. Catecholamines have been found to act through both β- and α-adrenergic receptors, which mediate their effects through different receptor pathways. Besides, many studies have demonstrated associations between β2 adrenergic receptor (β2-AR) gene polymorphisms and various diseases: obesity, type 2 diabetes (T2DM) [2], metabolic syndrome, metabolic disorders [3,4] and hypertension [5,6]. Therefore, the β2-AR gene may constitute a potential candidate gene to explain part of the genetic predisposition to human obesity and related traits.

Yang-Feng et al. reported that human β2-AR gene polymorphisms were located on the distal portion of the long arm of chromosome 5 (5q32-q34) [7]. Several polymorphisms have been found in the coding region of the β2-AR gene (Gly16Arg, Gln27Glu and Thr164Ile) and in the promoter region (5'LC-Cys19Arg), each of them leading to amino-acid substitution [8]. In their studies, Dallongeville et al. found that the Gly16Arg and Gln27Glu variants of the β2-AR gene contribute to metabolic syndrome susceptibility in men [4] Large et al. found that the Gln27Glu polymorphism of the β2-AR gene was markedly associated with obesity with a relatively higher risk for obesity and an odds ratio of approximately 10 [9]. The 5'LC-Cys19Arg protein modulates receptor translation [10], and Gly16Arg and Gln27Glu alterations of cellular trafficking and receptor desensitization [11-13] were found. Furthermore, another study [14] showed a significant association between the three polymorphisms of the β2-AR gene, 5'LC-Cys19Arg, Gly16Arg and Gln27Glu, and triglyceride (TG) and low-density lipoprotein cholesterol (LDL-C) plasma levels. So we hypothesized that the associations of these three single nucleotide polymorphisms (SNPs): 5'LC-Cys19Arg, Gly16Arg and Gln27Glu, and haplotypes of the β2-AR gene with lipid profiles, would be present in our study. In light of these considerations, we conducted a study in an ethnic Chinese elderly population to investigate the role of the three polymorphisms, 5'LC-Cys19Arg, Gly16Arg and Gln27Glu, of the β2-AR gene and related haplotypes in lipid profiles in elderly subjects.

The relationship between elevated serum total cholesterol (TC) and LDL-C concentrations, low HDL-C concentrations, and coronary heart disease in middle age [15-18] and early old age has been established [19]. In a past study [20], we established a possible module map among apolipoprotein E (Apo E), phospholipase A2 (PLA2) polymorphism, and lipid profiles in an elderly ethnic Chinese population. The presence of a physiologic balance contributed significantly to homeostasis and compensatory responses regulation of blood HDL-C and LDL-C profiles. In other past studies [21,22], we found the protective effect of the APoε2 allele against Alzheimer's disease and the higher risk of Alzheimer's disease in subjects with the APoε4 allele. And in another study [23], β-blockers were found to worsen delayed memory function in people with cognitive impairment, and adrenergic signaling was important for the retrieval of intermediate-term contextual and spatial memories. Furthermore, we thought that ApoE, PLA2 and β2-AR might all affect the lipid profiles and have interactions with each other, and associations with the causes of Alzheimer's disease. This study aimed to further explore the interactions between ApoE, PLA2 and β2-AR gene polymorphisms and lipid profiles in an elderly ethnic Chinese population.

## Materials and methods

### Participants

Employing a multilevel stratified sampling strategy, we selected subjects from the official household records of an entire prefecture in southern Taiwan. Three hierarchical household classifications below the county level were sequentially randomly sampled. A total of 500 healthy subjects without major medical diseases and mental illness aged older than 65 years was recruited for this study. There were 267 agreed to have venous blood drawn for DNA extraction for genetic study. In a past study [20], we explored the associations between ApoE, PLA2 and lipid profiles in these subjects. Informed consent was obtained, and the surveys were conducted by trained nurses.

### Data Collection and Laboratory Methods

All personal information was obtained during face-to-face interviews. Blood samples were obtained on the morning following a 12-hour fast. Cholesterol and triglyceride levels were determined in plasma, and lipoproteins by enzymatic methods. LDL-C was calculated using the equation of Friedewald et al. [24], and very-low-density lipoprotein cholesterol (VLDL-C) was derived from total cholesterol (TC) after subtracting LDL-C and HDL-C.

We characterized one SNP in the promoter region (5'LC-Cys19Arg) and two in the coding region (Gly16Arg and Gln27Glu) of the β2-AR gene. The missense mutations 5'LC-Cys19Arg, Gly16Arg and Gln27Glu of the β2-AR gene were genotyped using the flurogenic 5' nuclease assay application of the ABI PRISM 310 HT Sequence Detection System (ABI, Foster City, USA). Genotyping of the 5'LC-Cys19Arg was performed using primers (0.9 mmol/l each) Forward 5'-CCGCTGAATGAGGCTTCCA-3' and Reverse 5'-CCATGGCGCGCAGTCT-3' and the TaqMan MGB probes Fam TCAGCAGGCGGAC and Vic TCAGCGGGCGGAC. Genotyping of the Gly16Arg was performed using primers (0.9 mmol/l each) Forward 5'-GGCAGCGCCTTCTTGCT-3' and Reverse 5'-ACCCACACCTCGTCCCTTT-3' and the MGB probes Fam CCCAATGGAAGCCA and Vic CCCAATAGAAGCCATG. Genotyping of the Gln27Glu was performed using primers (0.9 mmol/l each) Forward 5'-GCGCCGGACCACGAC-3' and Reverse 5'-CCACCACCCACACCTCGT-3' and the MGB probes Fam TCACGCAGGAAAG and Vic TCACGCAGCAAAG.

Of the 10 ng/ml stok of DNA, 4 ml were dispensed into 384-well PCR plates using a Biomek FX robot (Beckman Coulter, Fullerton, USA), to which 6 ml of a mix containing the primers, MGB probes and TaqMan Universal PCR Master Mix (ABI, Foster City, CA, USA), were added as per the manufacturers' instructions. These were sealed with optical seals (ABI, Foster City, CA, USA) and incubated for 95°C 10 min, followed by 40 cycles of 95°C 15 s and 60°C 1 min before analysis on a 310HT plate reader (ABI, Foster City, CA, USA).

### Statistical Analysis

The outcomes identified were (1) TC; (2) HDL-C; (3) LDL-C; (4) VLDL-C; and (5) TG levels. Using an analysis of variance, we compared the average levels of plasma lipid among the different genotypes. Data were analyzed using the SPSS version 15.0 for Windows software package (Chicago, IL, U.S.A.). The unpaired t test was used for comparison between groups for continuous variables. Pearson's chi-square test was used for categorical variables. Hardy-Weinberg equilibrium was tested by means of gene counting and chi-square analysis. We used multiple linear regression models to examine the associations of serum lipids andβ2-AR gene polymorphisms. Analyses examined the lipid profile as continuous variables. Potential confounding variables (sex, age, education and income) were included. In addition, the Jump 5.1 for Windows software package was used to explore the interactive effect among β2-AR, PLA2 and Apo E polymorphism, sex, age and income with multivariate regression analysis for HDL-C and LDL-C. We explored the associations between haplotypes of theβ2-AR gene and lipid profiles by using Unphase Version 3.0 [25] and Phase version 2.1 [26] to code different haplotypes of theβ2-AR gene.

## Results

### Demographic Characteristics

Of the 500 subjects recruited in this study, 267 agreed to have venous blood drawn for DNA extraction for genetic study, yielding a response rate of 53.4%. The comparison of the socio-demographic characteristics of our respondents with data from the Ministry of the Interior, Taiwan, revealed that our survey sample did not substantially differ from the national sample [27]. The mean age was 69.2 years old in the case group (SD = 2.7), compared with 69.3 years old in the control group (SD = 2.8). The differences in mean age, sex, and monthly income were not statistically significant between the case and control group (p > 0.05). The case group included more educated persons than the control group (p = 0.013). The results are shown in Table 1.

**Table 1:** Comparison of Participating and Nonparticipating Subjects

**Variable**	**Participants (N = 267)**	**Nonparticipants (N = 233)**	P* Value
Age (yr) Mean (SDs)	69.18 (2.727)	69.18 (2.832)	0.990
Sex			0.148
Male (%)	157 (58.8)	122 (52.4)	
Female (%)	110 (41.2)	111 (47.6)	
Education			0.013
Illiterate (%)	117 (43.8)	128 (54.9)	
Primary school or above (%)	150 (56.2)	105 (45.1)	
Monthly income (USD)			0.107
<$860 (%)	193 (72.3)	190 (81.5)	
$860~2,000 (%)	59 (22.1)	33 (14.2)	
$2,001~2,860 (%)	12 (4.5)	8 (3.4)	
>$2,860 (%)	3 (1.1)	2 (0.9)	

*The *P *values for the percentages of sex, education, and monthly income refer to *X*2 tests of differences between participating and nonparticipating subjects. For age, the *P *value refers to the t-test of differences between participating and nonparticipating subjects.

### β2-AR Polymorphisms Genotypes Frequencies

The genotype frequencies of these three polymorphisms: 5'LC-Cys19Arg, Gly16Arg and Gln27Glu of β2-AR gene were all in Hardy-Weinberg equilibrium in the elderly ethnic Chinese population (5'LC-Cys19Arg Genotypes HWEχ ^2 ^= 03372, Gly16Arg Genotypes HWEχ^2 ^= 0.2135, Gln27Glu Genotypes HWEχ^2 ^= 0.5541, all < χ^2^_.95 _= 5.99, χ^2 ^in Hardy-Weinberg equilibrium [χ^2^_.95 _< 5.99, df = 2]). The genotype frequencies of these three polymorphisms are shown in Table 2, but the allele frequencies are not shown. In 5'LC-Cys19Arg polymorphisms, genotype Cys/Cys appeared most frequently (n = 256, 95.93%), followed by Cys/Arg (n = 10, 3.7%), and Arg/Arg (n = 1, 0.4%). In Gly16Arg polymorphisms, genotype Gly/Arg appeared most frequently (n = 118, 44.2%), followed by Arg/Arg (n = 85, 31.8%), and Gly/Gly (n = 46, 17.2%); 18 cases were undetectable. In Gln27Glu polymorphisms, Genotype Gln/Gln appeared most frequently (n = 247, 92.5%), followed by Gln/Glu (n = 19, 7.1%), and Glu/Glu (n = 1, 0.4%).

**Table 2:** β2-AR Genotype Frequencies and Plasma Lipids Among Genetically Unrelated Elderly Ethnic Chinese Individuals

**5'LC-Cys19Arg genotypes**				
Lipid level*	**Arg/Arg (n = 1)**	**Arg/Cys (n = 10)**	**Cys/Cys (n = 256)**	***P *Value**†

TC	282.0	200.0 (23.1)	215.15 (40.2)	0.129
HDL-C	47.0	45.3 (12.4)	51.13 (14.1)	0.422
LDL-C	204.0	129.7 (18.8)	137.42 (35.9)	0.138
VLDL-C	31.0	25.0 (10.5)	26.67 (15.4)	0.906
TG	151.0	127.0 (53.9)	133.52 (77.1)	0.940

**Gly16Arg genotypes**				

Lipid level*	**Arg/Arg (n = 85)**	**Gly/Arg (n = 118)**	**Gly/Gly (n = 46)**	***P Value*†**
TC	216.47 (40.862)	216.10 (41.5)	209.35 (39.2)	0.770
HDL-C	51.92 (14.588)	50.97 (14.8)	51.22 (12.3)	0.283
LDL-C	139.54 (36.410)	138.03 (34.9)	131.70 (36.5)	0.679
VLDL-C	25.01 (13.626)	27.28 (14.9)	26.43 (18.9)	0.512
TG	125.28 (68.208)	136.82 (74.7)	132.17 (94.4)	0.527

**Gln27Glu genotypes**				

Lipid level*	**Gln/Gln (n = 247)**	**Gln/Glu (n = 19)**	**Glu/Glu (n = 1)**	**P Value†**
TC	216.42 (40.287)	190.63 (35.7)	282.0	0.007
HDL-C	51.20 (14.276)	47.21 (11.1)	47.0	0.475
LDL-C	138.36 (35.378)	121.21 (33.0)	204.0	0.022
VLDL-C	26.95 (15.590)	22.21 (8.8)	31.0	0.410
TG	134.90 (78.011)	112.11 (45.3)	151.0	0.443

*Lipid level: mean (SDs) (mg/dL)

† The *P *values for all variables refer to F tests of differences between theβ2-AR genotype categories.

### β2-AR Gene Polymorphisms Association with Plasma Lipid Levels

Mean values of the overall plasma lipid levels in the elderly Chinese population across these three β2-AR gene polymorphism genotypes are shown in Table 2. The only two statistically significant differences were noted in TC and LDL-C in Gln27Glu of the β2-AR gene polymorphism (P = 0.007, P = 0.022). In 5'LC-Cys19Arg of the β2-AR gene polymorphism, Genotype Arg/Arg subjects had higher TC and LDL-C levels, but no statistically significant difference was noted (P = 0.129, P = 0.138), and only one subject had Genotype Arg/Arg. In the β2-AR Gly16Arg gene polymorphism, no significant difference was noted.

Using linear regression coefficients between the plasma lipid levels, the Gln27Glu polymorphisms of the β2-AR gene, Apo E and PLA2 genotypes, and other possibly related factors (e.g., sex, age, education, income) were summarized, as below (Table 3). Only females had lower TC levels, compared to males, with a statistically significant difference (p = 0.018). Although Gln27Glu polymorphisms Genotype Glu/Glu or Gln/Glu subjects had lower TC levels compared to Gln27Glu polymorphisms Genotype Gln/Gln subjects, no statistically significant difference was noted (p = 0.092). Furthermore, PLA2 A2 subjects had lower TC levels compared to Non-PLA2 A2 subjects, and TC levels trended downward with increased age, but neither showed a statistically significant difference (p = 0.057, p = 0.053). In HDL-C, the positive effects of the ε2 allele and female sex were statistically significantly (P = 0.032, p = 0.027). Age, PLA2 A2 allele effect and Glu effect on Gln27Glu polymorphisms in the β2-AR gene were not statistically significant. In LDL-C, the negative effects of the PLA2 A2 allele and age were significant (p = 0.037, p = 0.039). The ε2 allele effect, the Glu effect on Gln27Glu polymorphisms in the β2-AR gene, and female sex were not statistically significant.

**Table 3:** The Parsimonious Model for TC, HDL-C and LDL-C

	TC		HDL-C		LDL-C	
Variables	Coef (S.E.)	P	Coef (S.E.)	P	Coef (S.E.)	P
						
ε2allele effect	9.9(13.9)	0.474	11.0(5.1)	0.032	2.2(12.2)	0.860
A2 allele effect	-9.6(5.0)	0.057	1.3(1.8)	0.486	-9.3(4.4)	0.037
Glu effect	-16.1(9.5)	0.092	-2.9(3.5)	0.401	-8.2(8.4)	0.333
Sex	12.1(5.1)	0.018	4.2(1.9)	0.027	6.3(4.5)	0.164
Age	-1.8(0.9)	0.053	0.3(0.3)	0.442	-1.7(0.8)	0.039

ε2allele effect: Apoε2 vs. Non-Apoε2, A2 allele effect: PLA2 A2 vs. Non-PLA2 A2.

Glu effect: Genotype Glu/Glu, Gln/Glu vs. Genotype Gln/Gln in Gln27Glu Genotypes of β2-AR gene. Sex: female vs. male

TC: Total cholesterol; HDL-C: high-density lipoprotein cholesterol; LDL-C: Low-density lipoprotein cholesterol

Coef: Estimate coefficient; S.E.: Standard error; P: P value

### Income Associated with Plasma Lipid Levels

The 267 elderly subjects were then divided into two groups based on income: those with monthly income less than 860 USD (low income group; 193 subjects) and those with income of 860 USD or more (high income group; 74 subjects). We explored the different lipid profiles between the high income group and low-income group. In spite of the lack of statistically significant difference, the low-income group seemed to have a higher HDL-C level (standardized coefficient = 0.109, t = 1.781, p = 0.076, data not shown).

### Different Haplotypes of the β2-AR gene Associated with Plasma Lipid Levels

Based on the different genotypes of these three polymorphisms, 5'LC-Cys19Arg, Gly16Arg and Gln27Glu of the β2-AR gene, we had different haplotypes of the β2-AR gene. By using Unphase Version 3.0 analysis, we coded the different haplotypes of the β2-AR gene, and then explored the associations between the different haplotypes and lipid profiles of these elderly subjects. We found that only the Cys^19^Arg^16^Glu^27 ^haplotype and the Cys^19^Gly^16^Glu^27 ^haplotype in subjects had some association with lipid profiles. We found lower TC levels (p = 0.082) and lower LDL-C levels (p = 0.045) in subjects with the Cys^19^Arg^16^Glu^27 ^haplotype, and also lower TC levels (p = 0.06) in subjects with the Cys^19^Gly ^16^Glu^27 ^haplotype (Table 4). Only two subjects had the Cys^19^Arg^16^Glu^27 ^haplotype and six subjects, the Cys^19^Gly ^16^Glu^27 ^haplotype. We also found higher VLDL-C (P = 0.185) and higher TG levels (P = 0.190) in subjects with the Cys^19^Gly ^16^Gln^27 ^haplotype. However, the above results did not reach statistical significance.

**Table 4:** Plasma Lipids by Different β2-AR Gene Haplotypes Among Genetically Unrelated Elderly Chinese Individuals

Lipid level*	**Non-TGC**	**TGC**	**P Value**^†^
TC	214.69 (41.060)	214.66 (40.298)	0.994
HDL-C	51.11 (14.285)	50.74 (14.044)	0.841
LDL-C	138.64 (36.229)	136.49 (35.255)	0.638
VLDL-C	24.95 (13.279)	27.54 (16.246)	0.185
TG	125.03 (66.570)	137.88 (81.280)	0.190

Lipid level*	**Non-TGG**	**TGG**	**P Value**^†^

TC	215.38 (39.954)	184.00 (55.328)	0.060
HDL-C	50.98 (14.182)	46.33 (10.013)	0.426
LDL-C	137.68 (35.176)	119.50 (50.087)	0.216
VLDL-C	26.81 (15.377)	18.17 (5.456)	0.171
TG	134.27 (76.960)	90.17 (26.947)	0.163

Lipid level*	**Non-TAC**	**TAC**	**P Value**^†^

TC	209.82 (39.488)	215.66 (40.715	0.379
HDL-C	51.31 (12.387)	50.78 (14.455)	0.819
LDL-C	132.07 (36.841)	138.33 (35.276)	0.283
VLDL-C	26.44 (19.113)	26.65 (14.416)	0.936
TG	132.22 (95.497)	133.49(72.206)	0.919

Lipid level*	**Non-TAG**	**TAG**	**P Value**^†^

TC	215.05 (40.399)	165.00 (18.385)	0.082
HDL-C	50.81 (14.138)	59.50 (2.121)	0.386
LDL-C	137.65 (35.377)	87.00 (26.870)	0.045
VLDL-C	26.67 (15.311)	18.50 (6.364)	0.452
TG	133.57 (76.639)	94.50 (36.648)	0.473

Lipid level*	**Non-CGG**	**CGG**	**P Value**^†^

TC	214.98 (40.812)	207.45 (40.812)	0.547
HDL-C	51.11 (14.171)	45.45 (33.040)	0.194
LDL-C	137.30 (35.870)	136.45 (28.623)	0.939
VLDL-C	26.66 (15.470)	25.55 (10.152)	0.814
TG	133.45 (77.416)	129.18 (51.610)	0.856

*Lipid level: mean (SDs) (mg/dL); ^† ^The *P *values for all variables refer to T tests of differences between the two groups.

TGC: Cys^19^Gly^16^Gln^27^; TGG: Cys^19^Gly^16^Glu^2 ^; TAC: Cys^19^Arg^16^Gln; TAG: Cys^19^Arg^16^Glu^27^; CGG: Arg^19^Gly^16^Glu^27^

Using Phase Version 2.1 analysis to define the haplotypes of all subjects, we found that each subject in this study had two sequences of haplotypes of the β2-AR gene. We then divided these elderly subjects into two groups, according to whether they possessed the Cys^19^Gly ^16^Gln^27^haplotype or not. We could not find any statistically significant difference in lipid profiles between these two groups of elderly, using logistic regression analysis.

## Discussion

In our study, the ε2 allele and female sex had statistically significant (p = 0.032, p = 0.027) positive effects on HDL-C. The low income group also seemed to have higher HDL-C levels (P = 0.076). In LDL-C, the PLA2 A2 allele and age had significantly (p = 0.037, p = 0.039) negative effects. So, we explored the interactive effects among PLA2 and the Apo E polymorphism, sex and income on HDL-C, using multivariate regression analysis with the Jump 5.1 for Windows software package. We found that the ε2 allele combined with low income had a positive effect on HDL-C (p = 0.0011). We also found that the A2 allele combined with the ε2 allele had a positive effect on LDL-C (p = 0.0002).

In another study [14], Petrone et al. found that 5'LC-Cys^19 ^homozygous showed higher TG and LDL-C levels compared to 5'LC-Arg^19 ^homozygous. A similar increase in TG and LDL-C levels was observed for the Gly16Arg polymorphism and the Gln27Glu polymorphism. And, they found that the Cys^19^Arg^16^Gln^27 ^haplotype determined a significant increase in TG and LDL-C levels compared to the Arg^19^Gly^16^Glu^27 ^haplotype. After performing the final parsimonious model of multiple linear regression coefficients between the plasma lipid levels, the Gln27Glu polymorphism of the β2-AR gene, Apo E and PLA2 genotypes, and other possibly related factors, the Gln27Glu polymorphism Glu/Glu or Gln/Glu subjects had lower TC levels compared to the Gln27Glu polymorphism Gln/Gln subjects, but no statistically significant difference was noted (p = 0.092) (Table 3). And in different haplotypes of the β2-AR gene associated with plasma lipid levels, we found significantly lower TC levels (p = 0.082) and lower LDL-C levels (p = 0.045) in subjects with the Cys^19^Arg^16^Glu^27 ^haplotype, and significant lower TC levels (p = 0.06) in subjects with the Cys^19^Gly ^16^Glu^27 ^haplotype (Table 4). But there were too few subjects with these two haplotypes. We also found higher VLDL-C levels (p = 0.185) and higher TG levels (p = 0.190) in subjects with the Cys^19^Gly ^16^Gln^27 ^haplotype. Altogether, none of our results showed a statistically significant difference.

However, we believe that the difference in results between our study and the study by Petrone et al.^15 ^regarding the β2-AR gene may be due to the following reasons. Firstly, their subjects were overweight/obese subjects (body mass index [BMI] >25 kg/m^2^), who were consecutively recruited from the metabolic day hospital of the Department of Clinical Sciences of their university. Our subjects were selected from southern Taiwan, and were older than 65 years when they were recruited for this study. The difference in subjects between their study and our study may have led to the different results. And we had not let our subjects to check BMI in our study. So we can not discuss the impact of BMI on lipid profiles. On the other hand, we had not use case-control study to evaluate the different lipid profiles between obesity subjects and control cases. Secondly, our study had fewer subjects than theirs, and there were fewer frequencies of some polymorphisms of the β2-AR gene. The fewer subjects may have led to subjects with fewer of some polymorphisms of the β2-AR gene, thus leading to different results. In our study, the lipid profiles of the individual homozygous for the 5'LC-Arg19 allele and the lipid profile of the individual homozygous for the Glu27 allele are identical maybe due to this is the same individual. Thirdly, in their study, they only defined the frequency of each haplotype, and not the haplotypes of every subject. In our study, we used Phase Version 2.1 with Baysin analysis to define the haplotypes of all subjects to reduce the false positive rate [23].

On the other hand, we were also concerned that epistasis may present between multiple genes in lipid profiles. The low income group seemed to have a higher HDL-C level (p = 0.076) in our study, but in spite of this result not reaching statistically significant difference, we still wanted to add the income factor. After adding the income factor in the study, we found that the ε2 allele combined with low income had a positive effect on HDL-C (p = 0.0011). In our past study [20], we also found the opposite effects of the ε2 allele and the PLA2 A2 allele on HDL-C and LDL-C. The presence of the ε2 allele with the PLA2 A2 allele appeared to significantly decrease HDL-C, and the PLA2 A2 allele with the presence of the ε2 allele increased LDL-C.

## Conclusion

We concluded that not only do biological factors like Apo E, PLA2 and β2-AR affect lipid profiles, but social factors like income may also affect lipid profiles. We need to explore more of the effects that can affect lipid profiles to establish a clearer model to explain the interaction between genetic factors, social factors, and lipid profiles in the future. Using the model, if we can find high risk groups for hyperlipidemia and related disorders like metabolic syndrome, diabetes, cardiovascular disease, and obesity, and adopt adequate solutions, the prevalence of these disorders may be reduced. On the other hand, we may also have a clearer understanding of the causes of Alzheimer's disease using the model.

## Authors' contributions

FW had full access to all the design for this study, take responsibility for the integrity of the data and accuracy of the data analysis, and drafted the final manuscript, WT have carried out the molecular genetic studies, performed the statistical analysis, and drafted the manuscript, YC have conceived of the study, participated in its design and performed the statistical analysis. All authors read and approved the final manuscript.
